# Transcriptomic and iTRAQ-Based Quantitative Proteomic Analyses of *inap* CMS in *Brassica napus* L.

**DOI:** 10.3390/plants11192460

**Published:** 2022-09-21

**Authors:** Aifan Wang, Lei Kang, Guangsheng Yang, Zaiyun Li

**Affiliations:** 1 National Key Laboratory of Crop Genetic Improvement, National Center of Oil Crop Improvement (Wuhan), College of Plant Science and Technology, Huazhong Agricultural University, Wuhan 430070, China; 2 College of Agronomy, Hunan Agricultural University, Changsha 410128, China

**Keywords:** *Brassica napus*, cytoplasmic male sterility, transcriptome, iTRAQ, PCD, energy deficiency

## Abstract

*Brassica napus inap* cytoplasmic male sterility (CMS) is a novel sterile line with potential application in rapeseed hybrid breeding. Sterile cytoplasm was obtained from *Isatis indigotica* through somatic fusion and then recurrent backcrossing with *B. napus*. Previous studies have shown that *inap* CMS abortion occurred before the stamen primordia (stage 4–5), but the genetic mechanism of sterility needs to be studied. RNA-seq analyses were performed on the floral buds at two stages (0–5 and 6–8), before and after the formation of stamen primordium. As a result, a total of 1769 and 594 differentially expressed genes (DEGs) were detected in the CMS line compared to its maintainer line at the two stages, respectively. In accordance with the CMS phenotype, the up- and downstream regulators of the stamen identity genes *AP3* and *PI* were up- and downregulated in the CMS line, respectively. Furthermore, isobaric tags for relative and absolute quantitation (iTRAQ) analysis showed that a total of 760 differentially abundant proteins (DAPs) were identified in flower buds at stages 0–8, and most of the proteins related to the anther development, oxidative phosphorylation, and programmed cell death (PCD) were downregulated in *inap* CMS. In combined transcriptomic and proteomic analysis, a total of 32 DEGs/DAPs were identified, of which 7 common DEGs/DAPs had the same expression trend at stage 0–8 of flower development. The downregulation of genes related to the energy deficiency, hormone signal transduction, and the maintenance of mitochondrial metabolic homeostasis at stage 0–5 might disturb the normal differentiation of stamen primordium, resulting in carpelloid stamen of *inap* CMS. The study will help provide insights into the molecular mechanism of this new male sterility.

## 1. Introduction

Cytoplasmic male sterility (CMS) occurs naturally at a low frequency within the population but a quite widespread phenomenon among higher plant species. CMS is a type of male sterility caused by the interaction between nuclear and mitochondrial genomes [1]. CMS is not only widely used in many crops in producing hybrid seeds, but it is also a good model for nuclear–cytoplasm interaction research [2]. Many CMS genes (novel open reading frams, ORFs) result from rearrangements of the mitochondrial genome, and multiple mitochondrial genes have been cloned and verified in different plants species [3]. However, the study on the genetic molecular mechanism of plant male sterility is limited.

CMS-based breeding utilization is an effective way to develop novel rapeseed hybrid varieties of high-quality and high-yield. Although many *B. napus* CMS genes (*orf138* [4], *orf224* [5], *orf222* [6], *orf263* [7], and *orf346* [8]) have been cloned, the genetic and molecular basis of male sterility in *B. napus* remain to be further elucidated. In recent years, transcriptome and proteome analyses have been used to study the development of anthers and pollen related to male sterility in plants, such as rice [9], pepper [10], wheat [11], soybean [12], rapeseed [13], and broccoli [14]. Pollen development is a complex process, and many genes/proteins involved, programmed cell death (PCD) and energy conversion are specifically expressed in the CMS lines [15,16]. In Chinese cabbage, male sterility is related to the protein change in metabolic pathways, such as carbohydrate and energy metabolism, pollen wall synthesis and regulation, protein synthesis and degradation [16]. In *B. napus ogu* CMS, proteins involved in oxidative phosphorylation, the tricarboxylic acid (TCA) cycle, pollen wall, tetrad wall and PCD exhibited decreased accumulation in anther [17]. Abnormal β-1,3-glucosidase gene caused the corpus callosum to not be degraded as well as the male sterility in rice [18]. In addition, mineral nutrients, as the components of enzymes and electronic carriers, play an important role in the normal physiological and biochemical processes of plants. A certain concentration of Ca^2+^ in anthers is necessary for the development of anther and pollen. Too high or too low Ca^2+^ concentration may cause pollen abortion [19,20].

To avoid the potential threat of genetic vulnerability of rapeseed hybrids, it is of significance to explore and study a novel type of CMS system. The *inap* CMS of *B. napus* was developed from the somatic hybrid with *Isatis indigotica* (Chinese woad) by recurrent backcrossing [21]. Its tetradynamous stamens were carpelloid, and the two shorter stamens converted into filaments. The male sterility initiated at the stage of stamen primordium differentiation. The sterility of *inap* CMS was stable across different years and environments. The pistils of *inap* CMS displayed normal morphology and good seed-set after being pollinated by *B. napus*. The study showed that the *inap* CMS contained a mixed mitochondrial genome with both *B. napus* and *I. indigotica* mtDNAs but mostly from *I. indigotica*. Several chimeric genes were created by recombination (unpublished). The genetic mechanism of *inap* CMS sterility needs to be studied.

In the present study, we performed RNA-Seq and proteome profiling to identify the candidate pathways and genes related to male sterility in *inap* CMS. These differential expression proteins for male sterility were investigated for their potential mechanistic roles in anthers development and occurrence of male sterility in *inap* CMS. We hoped to obtain some specific differentially expressed genes or proteins related to the transformation of *inap* CMS stamens into carpel structures. The results may enhance our understanding of the genetic and molecular mechanism for CMS in *B. napus*.

## 2. Results

### 2.1. Phenotype of inap CMS and Its Maintainer

**Figure 1:**
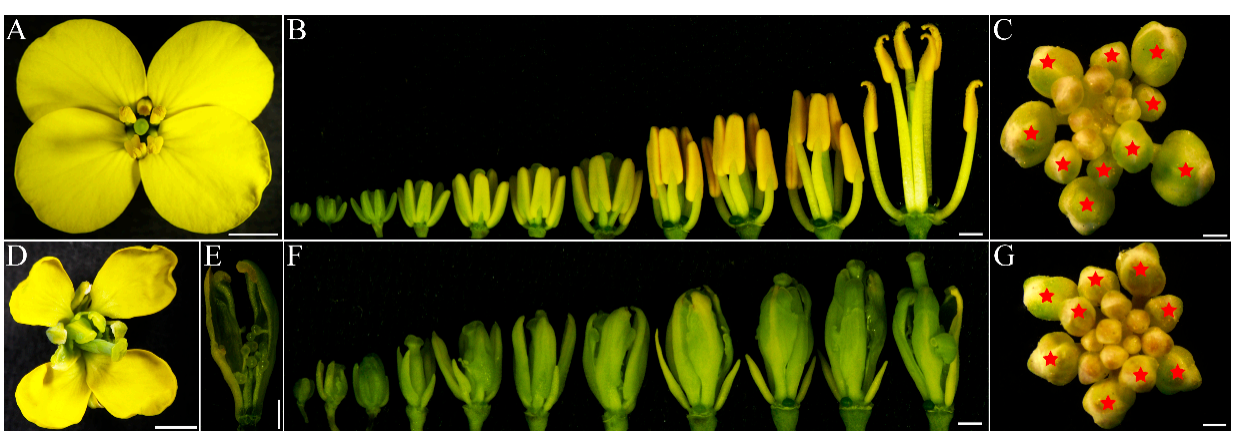
Flower morphology of *inap* CMS (**D**–**G**) and its maintainer “H3” (**A**–**C**) in *B. napus*. (**A**,**D**) Flowers; (**B**,**F**) Floral buds without sepals and petals. (**E**) Carpelloid stamen of *inap* CMS. (**C**,**G**) Flower buds at the stages 0–8. Red asterisks mark stage 6–8 buds. Bars: 1 mm.

The novel *inap* CMS has been backcrossed with the maintainer line Huashuang 3 (H3) more than 10 generations. There were no significant differences in morphology between *inap* CMS and H3, except for floral organs. The flowers and petals of *inap* CMS are smaller than those of H3 (Figure 1A,D), and the stamens of *inap* CMS transformed into carpels (Figure 1D–F). Based on the previous studies, the sterility of *inap* CMS occurred at the stage of stamen primordium differentiation (stage 5) [21]. Since then, the tetradynamous stamens of *inap* CMS transform into the carpel structures, with stigmatoid tissues at the tips and ovule-like tissues at the margins, and two shorter stamens into filaments (Figure 1B,E,F). As the CMS is regulated by mitochondria, we further investigated the mitochondrial structure in the carpelloid structures. From the observation of the ultrastructure of mitochondria in the cells of floral buds (stage 6–8) with transmission electron microscopy, compared with the maintainer line, the inner mitochondrial cristae disappeared in the *inap* CMS, the matrix concentration decreased, the shape was irregular, and the outer mitochondrial membrane was ruptured (Figure S1). Therefore, we collected the total floral buds at stages 0–5 (the bud length of H3 was less than 1.50 mm and that of *inap* CMS was less than 1.00 mm) and 6–8 (the flower buds of H3 were between 1.50 and 2.00 mm in length and those of *inap* CMS between 1.00 and 1.50 mm) for transcriptome analysis and total flower buds at stages 0–8 for proteomic analysis (Figure 1C,G).

### 2.2. Differential Expressed Genes Analysis

**Figure 2:**
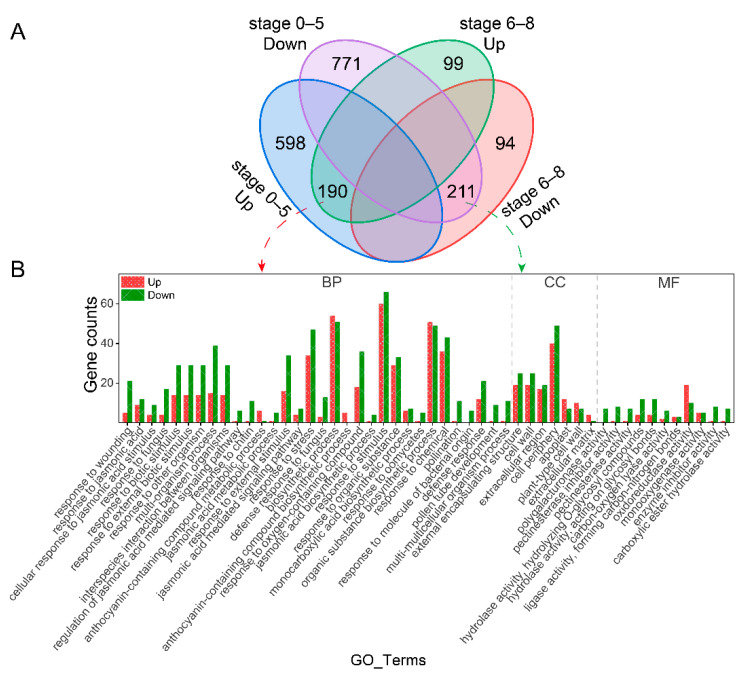
The analysis of DEGs. (**A**) Venn diagram of DEGs at stages 0–5 and 6–8 of flower development in *inap* CMS and H3; (**B**) GO annotation analysis of common DEGs at stages 0–5 and 6–8. Red and green bars represent up- and downregulated genes in *inap* CMS, respectively. BP, biological process. CC, cellular component. MF, molecular function.

RNA-seq analysis was performed on the total flower buds of *inap* CMS and H3 at two developmental stage sets (0–5 and 6–8). The mapping rates of all samples were above 87.5% (Table S1). The gene expression correlations between each pair of biological replicates were very high with all Pearson correlation coefficients (R^2^) > 0.996, except the sample H3 (stage 6–8) with the R^2^ = 0.887 (Figure S2). These results indicated that the sequencing data with high quality was suitable for subsequent analyses. Differentially expressed genes (DEGs) were identified with the threshold of |log_2_Fold change| ≥ 2 & Padj < 0.05. A total of 1770 DEGs (788 up- and 982 downregulated genes) were obtained at stage 0–5 of *inap* CMS compared with H3. At stage 6–8, 594 DEGs (289 up- and 305 downregulated genes) were obtained. Among these, 190 and 211 genes were upregulated and downregulated in both development stages, respectively (Figure 2A). These data provide a useful resource for exploring the molecular mechanism of *inap* CMS.

To explore the function of DEGs, we performed significant enrichment analysis of the DEGs with GO and KEGG (*p* < 0.05). One hundred and thirty-two significantly enriched GO terms were identified at stage 0–5 (*inap* CMS-vs-H3), including flavonoid biosynthetic process, pollen tube development, cell wall, oxidoreductase activity, pectinesterase activity, chalcone isomerase activity, hydrolase activity, and so on (Table S2). Among the DEGs at stage 6–8, there were 51 GO terms significantly enriched, such as cell wall, polygalacturonase activity, hydrolase activity, pectinesterase inhibitor activity, and pectinesterase activity (Table S2). GO significant enrichment analysis was also performed on the DEGs with the same expression trend at stages 0–5 and 6–8 (*inap* CMS-vs-H3), and all 190 up- and 211 downregulated genes were further classified into 50 GO terms. Only eight GO terms had more up-egulated genes than downregulated genes. Most genes involved in pollination, pollen tube development, polygalacturonase activity, pectinesterase activity, and hydrolase activity GO terms were downregulated in *inap* CMS (Figure 2B and Table S2).

**Figure 3:**
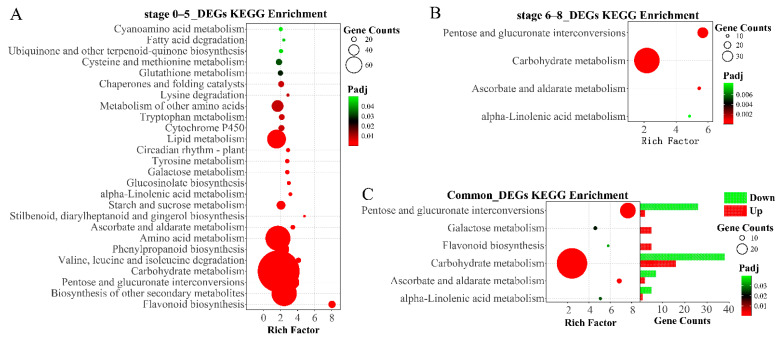
KEGG pathway significant enrichment analysis of DEGs at stage 0–5 (**A**), 6–8 (**B**), and both periods (**C**).

At stage 0–5 of the floral development, the DEGs were assigned to 26 KEGG pathways, mainly (23/26) classed into category of metabolism, including flavonoid biosynthesis, pentose and glucuronate interconversions, carbohydrate metabolism, amino acid metabolism, lipid metabolism, cytochrome P450, starch and sucrose metabolism, phenylpropanoid biosynthesis, etc. (Figure 3A and Table S2). At stage 6–8, 68 DEGs related to Carbohydrate metabolism, 29 DEGs involved in Pentose and glucuronate interconversions, 10 DEGs partook in Ascorbate and aldarate metabolism, and 8 DEGs associate with Alpha-Linolenic acid metabolism were obtained (Figure 3B and Table S2). The common DEGs in both two periods (0–5 and 6–8) were significantly enriched in six metabolic pathways (Figure 3C and Table S2). Among these, 38 and 26 genes related to Carbohydrate metabolism and Pentose and glucuronate interconversions, respectively, were downregulated in *inap* CMS, which might lead to insufficient energy supply and male sterility.

In addition to these DEGs that were significantly enriched in the GO and KEGG pathways, we also found some genes that may be related to stamen development. One gene *LEAFY* (*LFY*) was upregulated in *inap* CMS at stage 0–5 (Table S3). Six *AGAMOUS-like* (*AGL*) genes were identified, and two *AGL18* genes were downregulated in *inap* CMS at both stages (0–5 and 6–8). The *NAC-LIKE* activated by *AP3/PI* (*NAP*), involved in the transition between cell division and cell expansion during stamen development, was downregulated in *inap* CMS. One gene (*BnaA02g27790D*), which related to anther-specific protein *agp1-like*, was downregulated in *inap* CMS at stage 0–5. Later, at stage 6–8, the *REPRODUCTIVE MERISTEM 22* (*REM22*), which is expressed in stamen primordia, was downregulated in *inap* CMS. Furthermore, the *SPOROCYTELESS* (*SPL*), a key regulator of sporogenesis during stamen and carpel development, was downregulated in the CMS line (Table S3). These differentially expressed genes involved in stamen and carpel development were concordant with the CMS phenotype (Figure 1). Most genes which related to the NAC domain containing protein, cell wall invertase, pectate lyase protein, pectin methylesterase inhibitor, pectin methylesterase inhibitor, and *SKU5* similar were downregulated in *inap* CMS (Table S3).

### 2.3. Differential Abundant Proteins Analysis

To investigate the differential abundant proteins (DAPs) that were associated with abnormal stamen development in *inap* CMS lines, iTRAQ-based proteomic analysis was employed to assess protein changes between the buds of *inap* CMS and H3. A total of 406,338 secondary spectra were generated, of which 77,496 spectra were matched in the protein database, and 45,777 spectra were unique. Using “1% FDR” as a filtering criterion with Mascot, 18,919 unique peptides and 7954 proteins were identified between *inap* CMS and H3 (Figure S3A). The peptide number analysis displayed that a large portion of the identified proteins contained less than seven peptides, and more than 52% (4213) of the proteins identified one specific peptide, indicating that the protein was highly reliable (Figure S3B). The good coverage (the proportion > 5%) in the molecular mass ranged from 10 kDat o 80 kDa and >100 kDa, and the small molecular mass group (<10 kDa) had the fewest proteins (1.71%) (Figure S3C). The percentage of protein with peptide coverage of [0, 5%] was 25% (1964), [5%, 20%] peptide coverage accounted for 46% (3660) of the total proteins, while 29% (2330) of proteins’ peptide sequence coverage levels was greater than 20% (Figure S3D).

**Figure 4:**
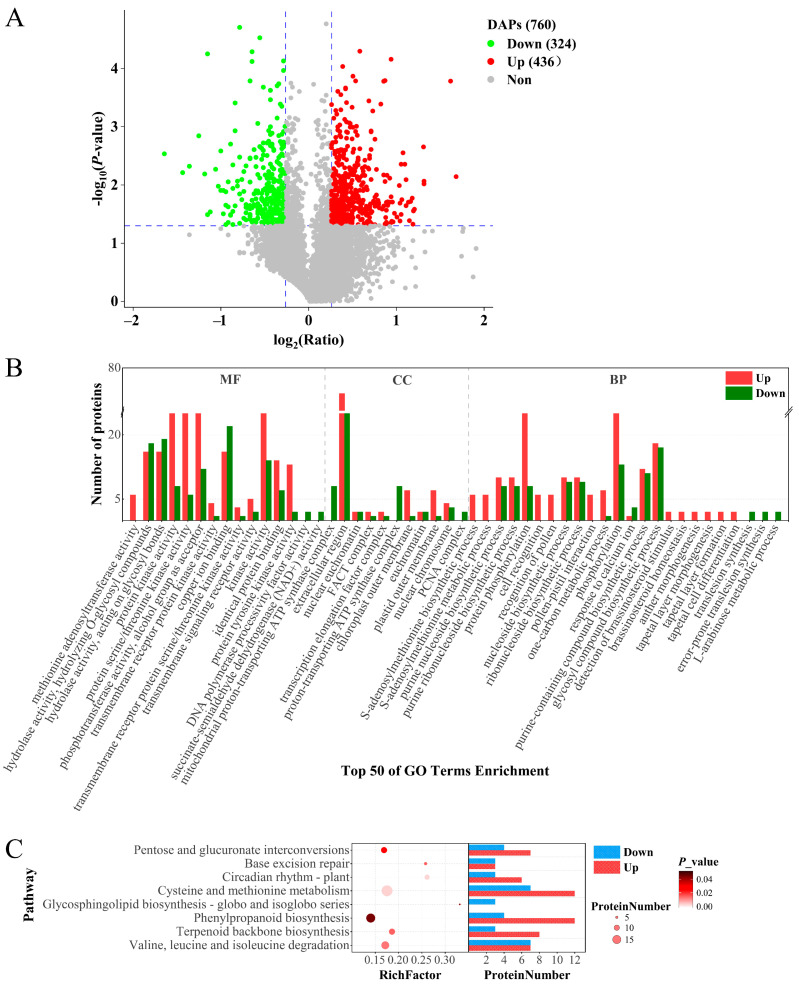
Analysis of differentially abundant proteins (DAPs) in floral buds of *inap* CMS line compared with its maintainer line H3. (**A**) Volcano plot of DAPs between *inap* CMS vs. H3. (**B**) The top 50 GO Terms with significant enrichment of DAPs. Red and green bars represent up- and downregulated proteins in *inap* CMS, respectively. MF, molecular function. CC, cellular component. BP, biological process. (**C**) KEGG significant enrichment analysis of DAPs. Red and blue bars represent up- and downregulated proteins in *inap* CMS, respectively.

Functional analysis of DAPs between *inap* CMS and H3 buds can provide valuable information for studying the sterility mechanism of the *inap* CMS in *B. napus*. We used CV values to assess quantitative reproducibility. The lower the CV value, the better the repeatability. In our study, the average variation in pairwise comparison groups between *inap* CMS and H3 was 19%, that is, CV = 19% (Figure S3E). The DAPs were identified using the restrictive thresholds of fold change ≥ 1.20, *p* value < 0.05, and unique peptide numbers ≥ 1. A total of 760 DAPs were identified, including 436 up- and 324 downregulated proteins in *inap* CMS compared with its maintainer lines H3 (Figure 4A). The most significantly enriched GO categories were displayed in Figure 4B. In terms of molecular function, “methionine adenosyl transferase activity” was the most significantly enriched with the DAPs all up-regulated. In addition, the DAPs involved in “DNA polymerase processivity factor activity” and “succinate-semialdehyde dehydrogenase (NAD^+^) activity” were all downregulated in *inap* CMS. All the downregulated proteins involved in “mitochondrial proton-transporting ATP synthase complex” belonged to cellular components. For the biological process ontology, “S-adenosyl methionine biosynthetic process” was the most significantly enriched, and the proteins were all upregulated in *inap* CMS.

To investigate the metabolic pathways in which the DAPs enriched, we performed KEGG pathway annotation analysis. As shown in Figure 4C, the DAPs were assigned to eight metabolic pathways, such as “Circadian rhythm-plant”, “Phenylpropanoid biosynthesis”, and “Pentose and glucuronate interconversions”, with most proteins being upregulated in *inap* CMS buds. All three proteins involved in “Glycosphingolipid biosynthesis-globo and isoglobo series” were downregulated in *inap* CMS. The DAPs in the different functional categories and metabolic pathways may provide a valuable resource for the study of male sterility in *inap* CMS.

### 2.4. DAPs Related to Male Sterility in inap CMS

Because the stamens of *inap* CMS were converted into carpeloid organs, we focus on the DAPs which related to anther and pollen development. As shown in Table 1, five proteins involved in carpel and ovule development were upregulated in *inap* CMS. We also obtained five proteins related to anther and pollen development which were downregulated in *inap* CMS. These results were consistent with the conversion of stamens into carpelloid structures of *inap* CMS, with ovule-like tissues at the margins (Figure 1). The previous study showed the disorganized cell proliferation of stamen primordium in *inap* CMS, resulting in the stamen converting into the carpelloid organ [21]. Pectinesterase, polygalacturonase, and beta-galactosidases were important in cell-wall modifications. In our study, two downregulated pectinesterases and polygalacturonases, two up- and three downregulated glycosyl hydrolases, and four up- and five downregulated beta-galactosidases were identified in *inap* CMS (Table 1 and Table S4). Changes in these cell-wall-remodeling proteins likely disturb the differentiation of the stamen primordium of *inap* CMS.

Mitochondria are the most important energy factories for the energy required for plant life activity. Seven DAPs associated with the oxidative phosphorylation pathway were downregulated in *inap* CMS, including two Acy1 carrier proteins, an accessory and non-catalytic subunit of the mitochondrial membrane respiratory chain NADH dehydrogenase (Complex I), which functioned in the transfer of electrons from NADH to the respiratory chain (Table 1). Five ATP synthase subunit proteins, mitochondrial membrane ATP synthase (F0F1-type ATP synthase) produce ATP from ADP in the presence of a proton gradient across the membrane, which is generated by electron transport complexes of the respiratory chain. These results indicate that the expression of these DAPs disturbed the normal mitochondrial morphology and function, leading to the male sterility in *inap* CMS.

Programmed cell death (PCD) is a phenomenon similar to apoptosis in plants, which is often driven by mitochondrial signals and participates in the development of plant organs. We found that eight DAPs associated with PCD were downregulated in *inap* CMS, including Glutathione S-transferase (GSTs) protein, persulfide dioxygenase protein, Thioredoxin (Trx) proteins, Peroxidases proteins, and Peroxiredoxin protein (Table 1). Downregulated expression of these proteins may lead to excessive accumulation of toxic compounds in the flower buds of *inap* CMS, resulting in male sterility.

### 2.5. Validation of DAPs by qRT-PCR Analysis

**Figure 5:**
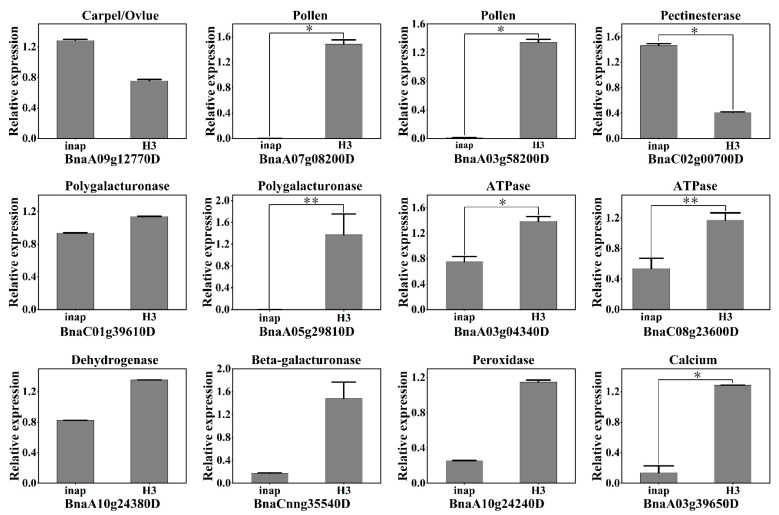
Real-time PCR confirmation of the relative expression of genes encoding 12 DAPs between *inap* CMS and its maintainer line (H3). All data are shown as the mean ± SD from three independent biological replicates. Significance at 0.01 < *p* < 0.05 level was marked with “*”; at *p* < 0.01 level was marked with “**”; while at *p* > 0.05 level was no marker which indicated no significant difference between *inap* CMS and H3.

To validate the iTRAQ data, we selected 12 DAPs that were potentially involved in male sterility for qRT-PCR assays (Figure 5; Table S5). These 11 DAPs which included pollen-specific protein-like, ATP synthase subunit O/d, D-lactate dehydrogenase [cytochrome], polygalacturonase, probable glucan endo-1, 3-beta-glucosidase A6, peroxidase, and calcium sensing receptor, showed similar expression patterns of those determined by iTRAQ. However, the BnaC02g00700D annotated as probable pectinesterase/pectinesterase inhibitor 51 displayed the opposite trend with the proteomic result, likely due to post-transcription translation. Taken together, these results were consistent with iTRAQ analysis, suggesting that the iTRAQ results were reliable.

### 2.6. Conjoint Analysis of DEGs and DAPs

**Figure 6:**
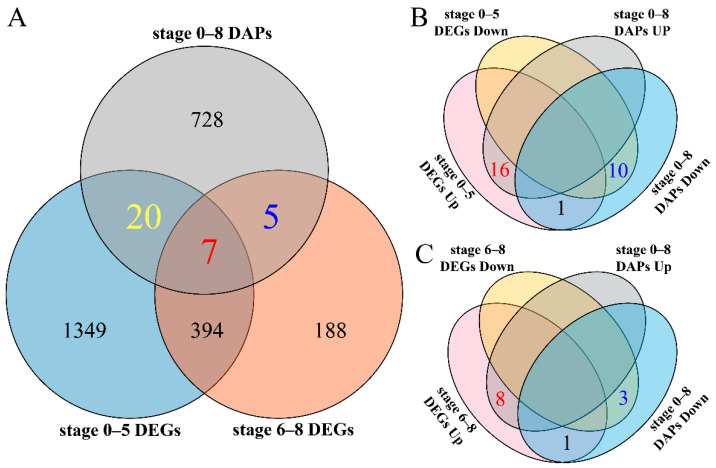
The Venn diagram of proteomic (iTRAQ) and transcriptomic (RNA-seq) analysis for the similarities and differences of DAPs/DEGs. (**A**) Conjoint analysis of proteins and transcripts showing unique and shared DAPs/DEGs. (**B**) and (**C**) up- and downregulated DAPs/DEGs at 0–5 and 6–8 periods in *inap* CMS, respectively.

To better understand and explore the mRNA and transcribed mRNA related to male sterility of *inap* CMS, a combined transcriptomic and proteomic analysis was performed. A total of 32 DEGs/DAPs were identified by both two methods, of which 7 common DEGs/DAPs had the same expression trend at stage 0–8 of flower development (Figure 6A, Table S6). Among them, six DEGs/DAPs related to peroxidase, chalcone synthase, and glycosyl hydrolase were upregulated in *inap* CMS, and the remaining one, which encoded Blue-copper-binding protein, was downregulated (Table S6). Compared with DEGs at stage 0–5, 26 DAPs (16 up- and 10 downregulated) showed the same expression trend, and 1 showed the opposite trend (Figure 6B). Nine up- and three downregulated DEGs/DAPs were obtained by the comparison of DAPs and DEGs at stage 6–8, and one DEGs/DAPs displayed an opposite trend (Figure 6C, Table S6). More importantly, at the earlier stage (0–5) of flower development, the downregulated DEGs/DAPs including acetyl-CoA acetyltransferase, aldehyde dehydrogenase, auxin-repressed protein, thioredoxin, and persulfide dioxygenase related to energy metabolism, hormone signal transduction, and maintaining mitochondrial metabolic homeostasis might disturb the normal differentiation of stamen primordium (Table S6).

## 3. Discussion

The *inap* CMS used in this study is a novel type of *B. napus* sterile line whose stamens are carpelized and can be inherited stably. Homeotic transformations of stamens into carpelloid organs have been reported in other CMS lines, such as wheat [22], tobacco [23], carrot [24], rapeseed [25], cauliflower [26], and broccoli [27]. Flowers of these CMS lines showed a phenotype similar to that of *A. thaliana* B-class mutants *AP3* and *PI*. It has been revealed that the B-class genes were downregulated in carpelloid CMS floral buds of tobacco, carrot, wheat, and rapeseed [22,23,28,29]. In this study, there was no significant reduction in the expression of *AP3*, *PI*, and *AG* genes in *inap* CMS compared to its maintainer line, with the log_2_ (fold change) scores ranging from −0.6 to −1.5 (Padj < 0.05). However, the *LFY* and *NAP* as the up- and downstream regulators of *AP3* and *PI* were up- and downregulated in *inap* CMS, respectively. Therefore, we speculate that the expression of the B-class genes in stamen were regulated by an as yet unknown factor, either directly or indirectly. Interestingly, three *AGAMOUS-like 18* (*AGL18*) genes were detected to be downregulated during floral bud development, and two of them (*BnaA09g36710D* and *BnaC08g28320D*) were downregulated at both development stages. The protein sequence is most similar to *AGL15* (*AGAMOUS-like 15*), which is expressed in developing embryos, and *AGL15* was also downregulated in stage 6–8 (Table S3). Unfortunately, we did not detect the relevant protein. In *B. juncea* CMS, the downregulated C-class gene *AG* resulted in the stamen’s petalization [30]. So, we speculate that the *AGL* gene may play an important role in stamen development. The *REPRODUCTIVE MERISTEM 22 (REM22)* reported to express in stamen primordia [31]. At the stage 6–8, two *REM22* were down-regulated in *inap* CMS. Therefore, the gene *REM22* may be significant for the carpel structures development of *inap* CMS. CMS is produced by the joint regulation of mitochondrial and nuclear genome, and four hypotheses have been proposed for the mechanisms that cause CMS: cytotoxicity, energy deficiency, PCD, and mitochondrial retrograde regulation [32].

As the second messenger of plants, Ca^2+^ is widely involved in regulating physiological and biochemical reactions in plants. It has been reported that the abnormal distribution Ca^2+^ in anthers may affect the development of pollen [20]. We obtained five DAPs (two up- and three downregulated) that might be related to the calcium ion transmembrane transport (Table S4). Therefore, we speculated that the abnormal distribution of Ca^2+^ in the buds of *inap* CMS would lead to disorders of intracellular physiological and biochemical metabolism and to male abortion.

### 3.1. PCD Related to Male Sterility in inap CMS

The CMS proteins which triggered PCD are related to the increase in reactive oxygen species (ROS), and peroxidase (POD) can prevent excessive accumulation of the ROS [33]. Glutathione sulfhydryl transferase (GST) also has POD activity, which can eliminate the toxicity of hydroxyperoxide [34]. We identified eight DAPs associated with PCD (Table 1). Among these DAPs, two peroxidase proteins (BnaA10g24240D and BnaA09g54380D) and one peroxiredoxin protein (BnaC08g24170D) were down-regulated in *inap* CMS. These three down-accumulated DAPs were all involved in peroxidase activity. This might lead to the over-accumulation of reactive oxygen in buds and the male sterility of *inap* CMS. The endogenous products of oxidative damage initiated by hydroxyl radicals are highly cytotoxic. Glutathione S-transferases (GSTs) have achieved detoxification by binding GSH with this endogenous electrophile. Some GSTs also act directly as glutathione peroxidases for detoxification [35]. In the glutathione metabolism pathway, downregulated glutathione S-transferase U13 (BnaA07g09120D) might cause the accumulation of toxic compounds in *inap* CMS. Thioredoxin (Trx) has various biological functions in keeping the stable redox status of cells. It has been reported that Trx in mitochondria maintained cell activity and plays a crucial role in the signal transduction process of apoptosis [36]. Trx is a physiological inhibitor of apoptosis-signal-regulated kinase-1 (ASK-1), which can inhibit cell apoptosis [37,38]. We identified three Trx DAPs (BnaC09g09340D, BnaC01g25910D, and BnaC08g42050D), which were downregulated in *inap* CMS. These downregulated enzymes may lead to the burst of ROS in *inap* CMS. Persulfide dioxygenase that plays an essential role in hydrogen sulfide catabolism in the mitochondrial matrix. High concentrations of sulfide can cause the instability of cytochrome oxidase [39]. The downregulated persulfide dioxygenase (BnaC03g69760D) may lead to the accumulation of toxic superphysiological H_2_S levels in *inap* CMS, which may destroy the metabolic stability of its mitochondria. The upregulation of a peroxidase and a thioredoxin protein in *inap* CMS may affect the accumulation of ROS (Table S4), and the imbalance of ROS can lead to male sterility. Therefore, PCD may be one of the causes of *inap* CMS male sterility.

### 3.2. Energy Metabolism Related to Male Sterility in inap CMS

A large amount of energy is necessary for the normal growth of flowering plants. Glycolysis, TCA cycle, oxidative phosphorylation, and other aerobic respiratory pathways are the main processes of ATP formation. ATP synthase is the key enzyme in the process of oxidative phosphorylation of mitochondria. The defect of mitochondrial F0F1-ATPase in pollen results in abnormal anther development [40].

**Figure 7:**
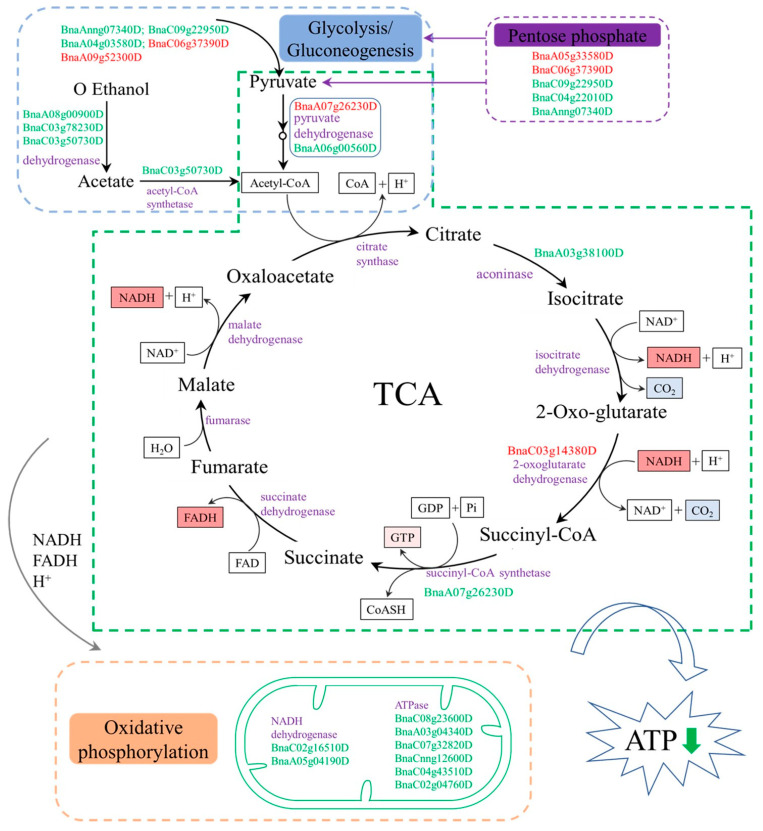
Differential abundance protein species (DAPs) involved in pathways related to carbohydrate and energy metabolism. Each pathway was surrounded by the dotted line in different colors. Purple words indicate the enzymes catalyzed in the relevant pathway. Red letters represent the upregulated and green letters represent the downregulated protein in *inap* CMS.

Most oxidation-reduction reactions in organisms are carried out under the catalysis of dehydrogenase and oxidase. In this study, we identified 10 DAPs related to dehydrogenases, and most (9) of the DAPs were downregulated in *inap* CMS flower buds, which may lead to energy deficiency and failure to meet the substantial energy requirement of stamen development. In addition, 12 DAPs (three up- and eight downregulated proteins) are related to the glycolysis/gluconeogenesis pathway (Figure 7). Two phosphofructokinase proteins and one transketolase protein associated with the pentose phosphate pathway were downregulated in *inap* CMS. This not only affects the normal progress of this pathway, but it also affects the production of substrates in the glycolysis and TCA cycle or reduces the concentration of the substrate. It has been reported that defects in the TCA cycle can lead to male sterility in rice [9]. As one of the important substrates of the TCA cycle, pyruvate participates in the glycolysis/gluconeogenesis pathway. In our study, five DAPs involved in TCA cycle, annotated as pyruvate dehydrogenase (BnaA06g00560D), aconinase (BnaA03g38100D), and succinyl-CoA synthetase (BnaA07g26230D), were downregulated in *inap* CMS. This may affect the conversion of oxaloacetic acid to phosphoenolpyruvate and cannot guarantee the function of the TCA cycle. The TCA cycle provides NADH, FADH, and H^+^ for oxidative phosphorylation. In this study, 11 DAPs were assigned to the oxidative phosphorylation pathway, 9 of which were downregulated in *inap* CMS buds. Furthermore, seven DAPs related to F0F1-ATPase were identified. The expression levels of these ATP synthase differential proteins in *inap* CMS flower buds were lower than those in maintainer flower buds (Figure 5 and Figure 7). What’s more, mitochondrial structures were disrupted in flower buds at stages 6–7 of *inap* CMS (Figure S1). Therefore, we speculate the mitochondrial dysfunction and energy deficiency in CMS lines, thereby affecting the normal development of stamens.

## 4. Materials and Methods

### 4.1. Plant Materials

The *inap* CMS line of *B. napus* and its maintainer line *cv*. Huashuang 3 were planted in Huazhong Agricultural University (Wuhan, China) experimental field under natural conditions. Based on the standards of development stages of *Arabidopsis* flower reported by Smyth [41], we accurately distinguished the development stages of rapeseed flowers under a stereo microscope. The total floral buds at stages 0–5 and 6–8 were used for RNA-seq analysis, respectively; since there were more proteins used for iTRAQ, the total floral buds at stages 0–8 were used for protein analysis (Figure 1C,G). There were only two biological replicates for each treatment due to material inaccessibility.

### 4.2. Transmission Electron Microscopy

The stamens of floral buds (stage 6–8) were fixed with 2.5% glutaraldehyde in PBS buffer and vacuumed until the sample sank to the bottom of the tube. After storage overnight at 4 °C, the samples were fixed with 1% osmic acid fixing solution at 4 °C for 2 h. After fixation, the samples were washed with PBS, dehydrated in a graded ethanol series, and embedded in Spurr’s resin. Ultra-thin sections were prepared using a Leica UC6 microtome (Leica, Weztlar, Germany) with a thickness of 50–70 nm. The sections were fished into a copper mesh and stained with 2% (*w*/*v*) uranyl acetate and 2.6% (*w*/*v*) lead citrate. The stained samples were observed using the Hitachi-7650 (Hitachi, Tokyo, Japan).

### 4.3. Transcriptome Analysis

The total RNA of floral buds was extracted using an RNAprep pure Plant Kit (TIANGEN, Beijing, China) following the manufacturer’s protocol. High-quality RNA (RIN ≥ 8) from each sample was used for cDNA library construction and RNA sequencing on an Illumina HiSeq^TM^ 3000 platform (Illumina) to generate 150 bp paired-end reads. Clean data (clean reads) were obtained by removing reads containing adapter and ploy-N, low-quality reads from raw data. All the downstream analyses were based on the clean data with high quality.

Clean reads of each sample were mapped to the *Brassica napus* reference genome (http://www.genoscope.cns.fr/brassicanapus/data/, accessed on 6 January 2022) with Hisat2 (version 2.0.5, University of Texas Southwestern Medical Center, Dallas, TX, USA) [42]. Gene expression levels were normalized using the FPKM (fragments per kilobase of transcript per million mapped reads) method [43] to define the differences in abundance expression gene between *inap* CMS and H3. The DESeq2 R package [44] was performed to identify differentially expressed genes (DEGs) between two samples. Corrected *p*-value (padj < 0.05) and |log_2_FlodChange| ≥ 2 as the threshold for significantly differential expression. Gene Ontology (GO) enrichment analysis of differentially expressed genes was implemented by the clusterProfiler R package, in which gene length bias was corrected. GO terms with corrected *p*-value < 0.05 were considered significantly enriched by differential expressed genes. DEGs pathway analysis was performed basing on the Kyoto Encyclopaedia of Genes and Genomes (KEGG) database (http://www.genome.jp/kegg/pathway.html, accessed on 6 January 2022). Pathways with Q-value < 0.05 were considered as significantly enriched pathways.

### 4.4. Protein Extraction and iTRAQ Analysis

Trichloroacetic acid–acetone-based method was used to extract the total protein of floral buds [45]. Then, four iTRAQ-labeled tags were used to label the samples. BP chromatography was conducted on a Shimadzu LC-20AB HPLC pump system. Through liquid phase separation of peptides were subjected to ESI tandem mass spectrometry analysis in a TripleTOF 5600 (SCIEX, Framingham, MA, USA).

The raw iTRAQ data files were converted to MGF files for protein species identification and quantification. Databases were searched by Mascot version 2.3.02 using the *Brassica_napus*. annotation v5 (https://www.genoscope.cns.fr/brassica-napus/data/, accessed on 9 September 2021), including 99,006 sequences. The IQuant [46] was performed for protein identification, which integrates the algorithm of Mascot Percolator [47] to improve the identification rate. We used CV values to assess quantitative reproducibility. CV = standard deviation/mean. The lower the CV value, the better the repeatability. All proteins with the false discovery rates (FDRs) ≤ 0.01 were filtered again to control the false positive rate of protein. Based on the criteria, fold-change thresholds of ≥1.2 (up-regulated) or ≤0.83 (downregulated), Q-value < 0.05, and unique peptide numbers ≥ 1 [48], the differentially abundant proteins (DAPs) between the *inap* CMS and its maintainer line H3 were identified.

All protein species identified were functionally annotated and classified into three ontologies (“molecular function”, “cellular component”, and “biological process”) based on Gene Ontology (GO) annotations (http://www.geneontology.org/, accessed on 9 September 2021). Protein pathway analysis was performed based on the Kyoto Encyclopedia of Genes and Genomes (KEGG) database (http://www.genome.jp/kegg/pathway.html, accessed on 9 September 2021). *p*-value < 0.05 was used as the threshold to further analyze DAPs using the GO and KEGG databases to identify significantly enriched functional subcategories and metabolic pathways.

### 4.5. Quantitative RT-PCR Analysis

We selected 12 DAPs for qRT-PCR assays, and all of them may be related to the anther development and male sterility in *inap* CMS of *B. napus*. The total RNA of floral buds was extracted using an RNAprep pure Plant Kit (Tiangen, DP441) following the manufacturer’s instructions. RNA was treated with RNase-free DNase I to remove genomic DNA. First-strand cDNA was synthesized using a RevertAid First Strand cDNA Synthesis Kit (Thermo Scientific, Waltham, MA, USA). Gene-specific primer sequences and detailed information are given in Table S3. qRT-PCR reactions were conducted using SYBR Green Realtime PCR Master Mix (Toyobo, Japan) with a CFX96 Touch Real-Time PCR Detection System (Bio-Rad, Hercules, CA, USA). The thermal cycler was performed as follows: 1 cycle of 95 °C 1 min; followed by 40 cycles of 95 °C for 10 s, 60 °C for 30 s, and 72 °C for 30 s. The 2^-ΔΔCt^ method [49] was used to analyze the results with CFX Manager software (Bio-Rad, Hercules, CA, USA). Three biological replicates (with three technical replicates for each biological replicate) were analyzed for each sample.

## 5. Conclusions

Through transcriptomic and proteomic analysis of floral buds to exploring the molecular mechanism of *inap* CMS of *B. napus*., we identified some DAPs/DEGs that were associated with phenylpropanoid biosynthesis, flavonoid biosynthesis, the TCA cycle, and oxidative phosphorylation pathways. Most of these DAPs/DEGs were downregulated in *inap* CMS, especially for some DAPs/DEGs related to peroxidase and ATP synthase, which might interfere with the pathways and lead to male sterility in *inap* CMS. This study lays the foundation for elucidating the molecular mechanism of *inap* CMS.

## Figures and Tables

**Table 1:** The main proteins involved in the occurrence of sterility in *inap* CMS.

DAPs_ID	Description	Mean_Ratio	*p*-Value	TAIR_ID
Carpel and ovule development				
BnaA09g12770D	Elongation factor G, chloroplastic	1.53	0.0206	AT1G62750
BnaA05g11640D	Transcription factor TCP10	1.43	0.0067	AT2G31070
BnaC06g12160D	O-acyltransferase WSD1	1.37	0.0196	AT5G37300
BnaC05g44320D	Arogenate dehydratase/prephenate dehydratase 2, chloroplastic	1.31	0.0001	AT3G07630
BnaC03g36730D	ABC transporter I family member 6, chloroplastic	1.29	0.0458	AT3G10670
Anther and pollen development				
BnaAnng09150D	Protein SHI RELATED SEQUENCE 5	0.74	0.0462	AT1G75520
BnaC05g00110D	Squamosa promoter-binding-like protein 8	0.72	0.0031	AT4G39400
BnaC01g09870D	Piriformospora indica-insensitive protein 2	0.56	0.0012	AT1G13230
BnaA07g08200D	Pollen-specific protein-like	0.48	0.0054	AT4G18596
BnaA03g58200D	Pollen-specific protein-like	0.37	0.0061	AT4G18596
Pectinesterase				
BnaC02g00700D	Probable pectinesterase/pectinesterase inhibitor 51	0.8	0.0004	AT5G09760
BnaA02g00180D	Probable pectinesterase/pectinesterase inhibitor 51	0.72	0.0419	AT5G09760
Polygalacturonase				
BnaA05g29810D	Exopolygalacturonase clone GBGA483	0.63	0.0002	AT3G07850
BnaA05g26870D	Polygalacturonase inhibitor 1	0.65	0.0142	AT5G06860
Oxidative phosphorylation				
BnaA05g04190D	Acyl carrier protein 1, mitochondrial	0.82	0.0247	AT2G44620
BnaC02g16510D	Acyl carrier protein 2, mitochondrial	0.81	0.0217	AT1G65290
BnaC02g04760D	ATP synthase subunit O, mitochondrial	0.7	0.0279	AT5G13450
BnaC04g43510D	ATP synthase subunit gamma, mitochondrial	0.68	0.0232	AT2G33040
BnaC07g32820D	ATP synthase subunit d, mitochondrial	0.67	0.0311	AT3G52300
BnaA03g04340D	ATP synthase subunit O, mitochondrial	0.56	0.0229	AT5G13450
BnaC08g23600D	ATP synthase subunit d, mitochondrial	0.53	0.0239	AT3G52300
PCD				
BnaA07g09120D	Glutathione S-transferase U13	0.75	0.0199	AT1G27130
BnaC03g69760D	Persulfide dioxygenase ETHE1 homolog, mitochondrial	0.75	0.0050	AT1G53580
BnaC09g09340D	Thioredoxin reductase 2	0.72	0.0176	AT2G17420
BnaC01g25910D	Thioredoxin M3, chloroplastic	0.68	0.0022	AT2G15570
BnaC08g42050D	Thioredoxin-like protein CXXS1	0.52	0.0131	AT1G11530
BnaA09g54380D	Peroxidase 22	0.75	0.0320	AT2G38380
BnaA10g24240D	Peroxidase 53	0.61	0.0217	AT5G06720
BnaC08g24170D	Peroxiredoxin-2E, chloroplastic	0.74	0.0111	AT3G52960
In order of Mean_Ratio (*inap*-vs-H3), and the DAPs were identified at level of *p*-value < 0.05.				

## Data Availability

Transcriptome data are accessible under NCBI BioProject no. PRJNA826967. The mass spectrometry proteomics data have been deposited to the ProteomeXchange Consortium (http://proteomecentral.proteomexchange.org) with the dataset identifier PXD035086.

## Supplementary Materials

The following supporting information can be downloaded at: https://www.mdpi.com/article/10.3390/plants11192460/s1, Figure S1: TEM detection of stage 6–7 floral buds; Figure S2: Pearson correlation coefficients between each pair of biological replicates under same sampling conditions; Figure S3: Proteomics and analyses of DAPs from *inap* CMS and its maintainer line H3; Table S1: Statistics of mapped reads; Table S2: The GO and KEGG annotations of DEGs between *inap* CMS and H3; Table S3: The main genes may be involved in the occurrence of *inap* CMS sterility; Table S4: The main proteins may be involved in the occurrence of *inap* CMS sterility; Table S5: The corresponding primers of qRT-PCR; Table S6: The common DEGs/DAPs expression in *inap* CMS sterile line.
