# Supplementary material for: Transcriptomic and iTRAQ-Based Quantitative Proteomic Analyses of inap CMS in Brassica napus L
Source: Plants (Basel). 2022 Sep 21;11(19):2460. doi: 10.3390/plants11192460 (PMC9571993; doi:10.3390/plants11192460)
Supplement: Supplementary file 1 [file plants-11-02460-s001.zip › Supplementary Figures.pdf]

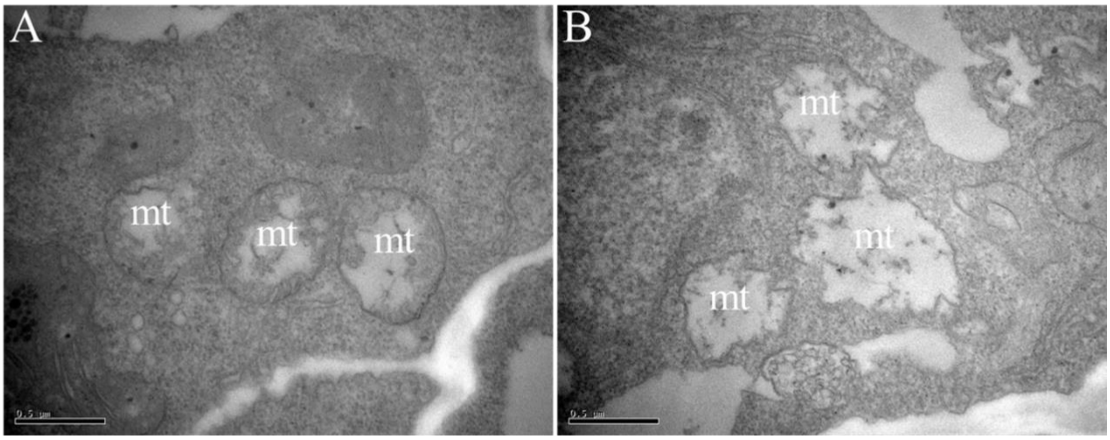

Figure S1. TEM detection of stage 6–8 floral buds. (**A**) Maintainer line (H3). (**B**) *inap* CMS sterile line. mt, mitochondria.

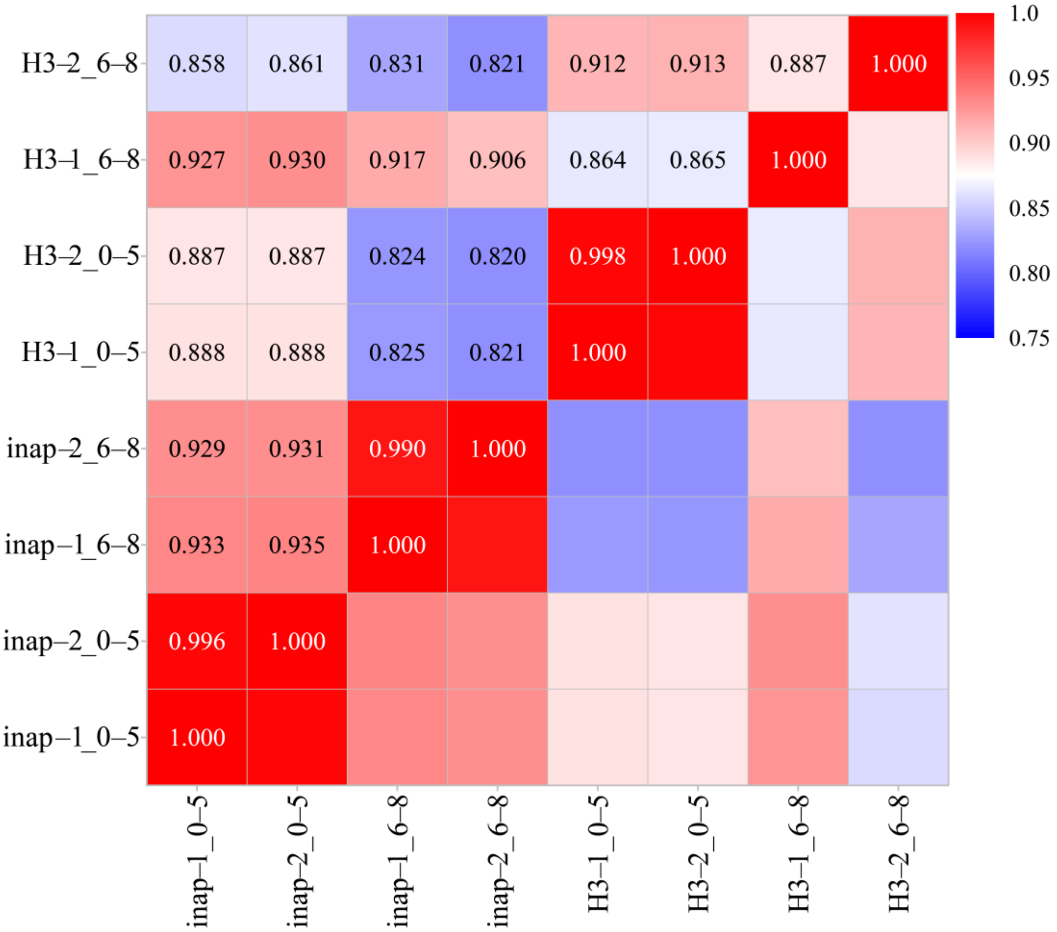

Figure S2. Pearson correlation coefficients between each pair of biological replicates under same sampling conditions.

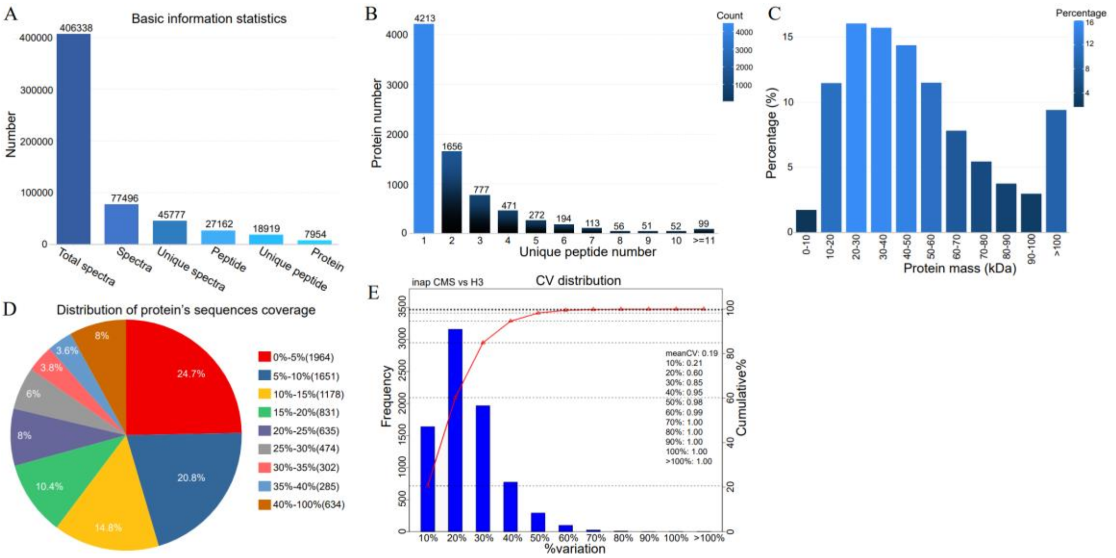

Figure S3. Proteomics analysis of floral buds at stage 0–8 in comparison between *inap* CMS and H3. (**A**) Number of spectra, peptide and protein in the iTRAQ analysis. Total spectra are the total number of secondary spectra, spectra indicated the number of matched spectra. (**B**) Number of peptides that match to unique proteins number. (**C**) Mass distribution of the predicted proteins. (**D**) Distribution of the proteins sequence coverage. The pie chart displays the percentage of proteins in the different ranges and the total protein numbers. The colors represent different coverage ranges of the protein sequence. (**E**) Analysis of reproducibility of the biological replicates in the two samples.
