# Supplementary material for: Transcriptomic and iTRAQ-Based Quantitative Proteomic Analyses of inap CMS in Brassica napus L
Source: Plants (Basel). 2022 Sep 21;11(19):2460. doi: 10.3390/plants11192460 (PMC9571993; doi:10.3390/plants11192460)
Supplement: Supplementary file 1 [file plants-11-02460-s001.zip › Supplementary Tables.pdf]

Table S1. Statistics of RNA-seq reads and mapped reads

Samples	Total clean reads	Mapping rates (%)
**inap–1 0–5**	32060818	89
**inap–2 0–5**	32956722	88.66
**inap–1 6–8**	51566905	88.05
**inap–2 6–8**	28754207	87.9
**H3–1 0–5**	32349064	88.91
**H3–1 0–5**	31953791	88.47
**H3–2 6–8**	37760788	87.59
**H3–2 6–8**	29438485	87.57

Table S3. The main genes that may be involved in the occurrence of sterility were identified from the *inap* CMS and H3 maintainer line.

Genes ID	Description	$\log_2$(Fold Change) (Stage 0–5)	$\log_2$(Fold Change) (Stage 6–8)	TAIR ID
BnaCnng78420D	Floral meristem identity control protein LEAFY (LFY)	2.422885432		AT5G61850
BnaC02g41870D	AGAMOUS-like 8 (AGL8)	4.232514048	3.056685218	AT5G60910
BnaA09g36710D	AGAMOUS-like 18 (AGL18)	-2.163229327	-2.779705885	AT3G57390
BnaC08g28320D	AGAMOUS-like 18 (AGL18)	-3.067970231	-2.255123355	AT3G57390
BnaC04g24260D	AGAMOUS-like 18 (AGL18)		-2.128139777	AT3G57390
BnaA02g33230D	AGAMOUS-like 42 (AGL42)		2.118124176	AT5G62165
BnaA03g04490D	AGAMOUS-like 15 (AGL15)		-2.017817741	AT5G13790
BnaC06g43410D	NAC-like, activated by AP3/PI (NAP)	-2.972590524		AT1G69490
BnaA07g24270D	NAC-like, activated by AP3/PI (NAP)	-2.215891592		AT1G69490
BnaA02g27790D	Anther-specific protein agp1-like	-2.035942335		AT3G26110
BnaA03g34370D	REPRODUCTIVE MERISTEM 22 (REM22)		-2.579005175	AT3G17010
BnaC03g39810D	REPRODUCTIVE MERISTEM 22 (REM22)		-2.4493508	AT3G17010
BnaA01g16350D	Sporocyteless (SPL)		-2.521612076	AT4G27330
BnaA03g58770D	Sporocyteless (SPL)		-2.273052532	AT4G27330
BnaC02g42720D	NAC domain containing protein 102	2.829477416		AT5G63790
BnaC03g50150D	NAC domain containing protein 103	2.49698767		AT5G64060
BnaA06g23210D	NAC domain containing protein 103	2.851585785		AT5G64060
BnaC06g05920D	NAC domain containing protein 19	-2.477049138		AT1G52890
BnaC06g05910D	NAC domain containing protein 19	-2.22022182		AT1G52890
BnaC06g05930D	NAC domain containing protein 19	-2.114869872		AT1G52890
BnaA05g24050D	NAC domain containing protein 3	-3.920059147	-2.416046987	AT3G15500
BnaC05g38150D	NAC domain containing protein 3	-3.323233529		AT3G15500
BnaC01g36040D	NAC domain containing protein 3	-2.925168843		AT3G15500
BnaA01g28930D	NAC domain containing protein 3	-2.237260187		AT3G15500
BnaC03g39160D	NAC domain containing protein 3		-2.361383318	AT3G15500
BnaA02g18390D	NAC domain containing protein 32	-3.192421125		AT1G77450
BnaC04g43920D	NAC domain containing protein 41	2.09582245		AT2G33480
BnaC03g58370D	NAC domain containing protein 5	-3.060329332	-2.644144979	AT1G02250
BnaC01g23890D	NAC domain containing protein 60	-2.096335244		AT3G44290
BnaA03g08460D	NAC domain containing protein 89	-3.394958545		AT5G22290

Genes ID	Description	log_2(Fold Change) (Stage 0–5)	log_2(Fold Change) (Stage 6–8)	TAIR ID
BnaC04g28060D	Cell wall invertase 2	-5.367356147		AT3G52600
BnaA04g05320D	Cell wall invertase 2	-4.693923179		AT3G52600
BnaC03g03990D	Cellulose synthase 5	2.938271608	2.112034338	AT5G09870
BnaC02g43280D	Cellulose synthase 6	4.424789884	3.115007843	AT5G64740
BnaC03g07610D	Cellulose synthase like A11		3.106490904	AT5G16190
BnaA04g18860D	Cellulose synthase-like B3		2.283646999	AT2G32530
BnaC04g11500D	Cellulose synthase-like D1	-4.307104236	-3.26897208	AT2G33100
BnaA05g10500D	Cellulose synthase-like D1	-4.93043586	-2.974262713	AT2G33100
BnaA03g27540D	Pectate lyase family protein	-3.778280517	-3.836891619	AT3G01270
BnaC03g32550D	Pectate lyase family protein	-4.869386448	-4.746838834	AT3G01270
BnaCnng05770D	Pectate lyase family protein	-5.732177227	-4.642224073	AT3G01270
BnaA06g09240D	Pectate lyase family protein	-5.752539104	-4.790405111	AT1G14420
BnaA08g23970D	Pectate lyase family protein	-5.5734459		AT1G14420
BnaA09g57110D	Pectate lyase family protein	-3.402143484		AT1G14420
BnaC05g10650D	Pectate lyase family protein	-3.532419932	-4.106068217	AT1G14420
BnaCnng60280D	Pectate lyase family protein	-6.295459974	-4.025722693	AT1G14420
BnaA02g13340D	Pectate lyase family protein	6.100979107	3.739166317	AT1G67750
BnaA07g38610D	Pectate lyase family protein	2.010340399		AT1G67750
BnaC06g26450D	Pectate lyase family protein	2.12949726		AT1G67750
BnaA09g19110D	Pectate lyase family protein	-4.741376147		AT2G02720
BnaC02g34750D	Pectate lyase family protein	-3.088415711		AT2G02720
BnaA02g26540D	Pectate lyase family protein		-3.025813932	AT2G02720
BnaC03g06740D	Pectate lyase family protein	-2.911216015		AT5G15110
BnaC07g49180D	Pectin lyase-like superfamily protein	-3.590797431	-5.273654671	AT5G48140
BnaC02g38360D	Pectin lyase-like superfamily protein	-4.350583362		AT5G48140
BnaCnng71300D	Pectin lyase-like superfamily protein	-2.367164417		AT5G48140
BnaA06g30420D	Pectin lyase-like superfamily protein	-3.939181219	-4.869246187	AT5G48140
BnaA03g30180D	Pectin lyase-like superfamily protein	-6.976506578	-4.723351973	AT3G07820
BnaC03g35490D	Pectin lyase-like superfamily protein	-5.95358812	-4.886626183	AT3G07820
BnaA03g34410D	Pectin lyase-like superfamily protein	-4.178772034	-3.486297539	AT3G17060
BnaC03g39840D	Pectin lyase-like superfamily protein		-3.755300512	AT3G17060

Genes ID	Description	log_2(Fold Change) (Stage 0–5)	log_2(Fold Change) (Stage 6–8)	TAIR ID
BnaA10g23870D	Pectin lyase-like superfamily protein	-3.265140545	-3.167913772	AT5G07420
BnaAnng13710D	Pectin lyase-like superfamily protein	-4.869934432		AT5G07430
BnaC03g03040D	Pectin lyase-like superfamily protein	-3.095143279		AT5G07430
BnaA03g02030D	Pectin lyase-like superfamily protein	-4.225350425		AT5G07430
BnaA10g23860D	Pectin lyase-like superfamily protein	-3.144098861		AT1G69940
BnaA04g13950D	Pectin lyase-like superfamily protein	-2.834078822		AT2G23900
BnaC04g36920D	Pectin lyase-like superfamily protein	-2.215811826		AT2G23900
BnaA08g03570D	Pectin methylesterase inhibitor 1	-4.85958817	-2.478582113	AT1G48020
BnaC08g03920D	Pectin methylesterase inhibitor 1	-2.303159602	-2.438977969	AT1G48020
BnaC03g35940D	Pectinacetylesterase family protein	2.304989484		AT3G09410
BnaC04g00460D	Plant invertase/pectin methylesterase inhibitor superfamily	-6.102750562	-5.965643044	AT2G47040
BnaC03g72420D	Plant invertase/pectin methylesterase inhibitor superfamily	-6.439175372	-5.576824961	AT2G47040
BnaA03g21590D	Plant invertase/pectin methylesterase inhibitor superfamily	-5.671898519	-5.322312634	AT2G47040
BnaA05g00810D	Plant invertase/pectin methylesterase inhibitor superfamily	-3.601395582	-2.676162028	AT2G47040
BnaCnng76410D	Plant invertase/pectin methylesterase inhibitor superfamily	-7.506107772		AT2G47040
BnaA03g21600D	Plant invertase/pectin methylesterase inhibitor superfamily	-5.246213818	-2.449865139	AT2G47050
BnaA04g26890D	Plant invertase/pectin methylesterase inhibitor superfamily	-5.672368939	-3.877372715	AT2G47050
BnaA05g00800D	Plant invertase/pectin methylesterase inhibitor superfamily	-5.342179271	-3.545318833	AT2G47050
BnaC03g72430D	Plant invertase/pectin methylesterase inhibitor superfamily	-4.715821267	-2.223685475	AT2G47050
BnaC04g00440D	Plant invertase/pectin methylesterase inhibitor superfamily	-3.102310853	-2.834306908	AT2G47050
BnaC04g51500D	Plant invertase/pectin methylesterase inhibitor superfamily	-5.415078998	-3.536789256	AT2G47050
BnaA02g30620D	Plant invertase/pectin methylesterase inhibitor superfamily	-6.132894432	-5.235602479	AT5G49180
BnaCnng75130D	Plant invertase/pectin methylesterase inhibitor superfamily	-5.560547779	-3.479528155	AT5G49180
BnaC03g34350D	Plant invertase/pectin methylesterase inhibitor superfamily	-3.798651411	-3.443127276	AT3G05610
BnaA03g29100D	Plant invertase/pectin methylesterase inhibitor superfamily	-5.252449814	-3.092526921	AT3G05610
BnaC03g03910D	Plant invertase/pectin methylesterase inhibitor superfamily	4.787842609	3.231611726	AT5G09760
BnaC09g50270D	Plant invertase/pectin methylesterase inhibitor superfamily	3.538787103		AT5G04970
BnaA03g22710D	Plant invertase/pectin methylesterase inhibitor superfamily	-5.339904456	-2.792347273	AT2G26450
BnaC03g26740D	Plant invertase/pectin methylesterase inhibitor superfamily	-4.67830567		AT2G26450
BnaA01g32580D	Plant invertase/pectin methylesterase inhibitor superfamily	-2.251754798		AT3G06830
BnaC01g16500D	Plant invertase/pectin methylesterase inhibitor superfamily protein	-5.108883119	-3.904508418	AT4G24640

Genes ID	Description	$\log_2$(Fold Change) (Stage 0–5)	$\log_2$(Fold Change) (Stage 6–8)	TAIR ID
BnaA01g14010D	Plant invertase/pectin methylesterase inhibitor superfamily protein	-5.964558561	-3.109305573	AT4G24640
BnaA06g06610D	Plant invertase/pectin methylesterase inhibitor superfamily protein	-4.748646549	-2.41329798	AT1G10770
BnaA09g47760D	Plant invertase/pectin methylesterase inhibitor superfamily protein	-2.857821644		AT1G10770
BnaC05g08260D	Plant invertase/pectin methylesterase inhibitor superfamily protein		-2.049038167	AT1G10770
BnaA02g33370D	Plant invertase/pectin methylesterase inhibitor superfamily protein	2.287802724		AT5G62350
BnaA09g00690D	Plant invertase/pectin methylesterase inhibitor superfamily protein	-3.957710589		AT4G02250
BnaCnng52530D	Plant invertase/pectin methylesterase inhibitor superfamily protein	-3.050811037		AT1G23350
BnaCnng57640D	Plant invertase/pectin methylesterase inhibitor superfamily protein	-2.758217485		AT3G62180
BnaA04g27250D	Plant invertase/pectin methylesterase inhibitor superfamily protein	-2.011299789		AT3G62180
BnaC06g36860D	SKU5 similar 5	2.073564907		AT1G76160
BnaA05g25900D	SKU5 similar 12		-3.445459845	AT1G55570
BnaC05g39980D	SKU5 similar 12	-4.653275895		AT1G55570
BnaC03g37900D	SKU5 similar 12	-2.958159879		AT1G55570
BnaA03g32810D	SKU5 similar 13	-6.139936772	-5.642868991	AT3G13400
BnaA05g25890D	SKU5 similar 13	-7.031067839	-5.168883918	AT3G13400
BnaC01g37680D	SKU5 similar 13	-3.807186359	-5.139840421	AT3G13400
BnaA01g29830D	SKU5 similar 13	-5.287851797	-5.08860411	AT3G13400
BnaC05g39970D	SKU5 similar 13	-6.733978967	-4.924595037	AT3G13400
BnaC03g37910D	SKU5 similar 13	-6.885571999		AT3G13400
BnaC03g70650D	SKU5 similar 14	-2.580045603	-3.65879561	AT1G55560

Table S4. The main proteins that may be involved in the occurrence of sterility were identified from the *inap* CMS and H3 maintainer line.

DAPs ID	Description	Mean Ratio	*P*-value	TAIR ID
Glycosyl hydrolases				
BnaC09g12230D	Endoglucanase 6	1.83	0.0001622	AT1G64390
BnaA02g17370D	Endoglucanase 10	1.42	0.01416	AT1G75680
BnaC04g08890D	1,4-alpha-glucan-branching enzyme 2-1, chloroplastic/amyloplastic	0.83	0.006174	AT2G36390
BnaCnng30450D	Beta-D-xylosidase 4	0.76	0.007854	AT5G64570
BnaAnng23050D	Xyloglucan endotransglucosylase/hydrolase protein 24	0.68	0.007605	AT4G30270
Beta-galactosidases				
BnaC08g28150D	Glucan endo-1, 3-beta-glucosidase, acidic isoform	1.61	0.02594	AT3G57260
BnaA05g14890D	Beta-D-glucopyranosyl abscisate beta-glucosidase	1.48	0.003734	AT1G52400
BnaC06g13950D	Glucan endo-1, 3-beta-glucosidase 10	1.42	0.01481	AT5G42100
BnaC05g06540D	Mannosylglycoprotein endo-beta-mannosidase	0.82	0.04935	AT1G09010
BnaA10g11860D	Glucan endo-1, 3-beta-glucosidase 9	0.82	0.02535	AT5G58480
BnaA01g34050D	Probable inactive beta-glucosidase 25	0.75	0.001293	AT3G03640
BnaC04g29090D	Probable glucan endo-1, 3-beta-glucosidase A6	0.58	0.003338	AT4G14080
BnaCnng35540D	Probable glucan endo-1, 3-beta-glucosidase A6	0.57	0.01051	AT4G14080
PCD				
BnaC01g31810D	Peroxidase 30	1.75	0.02477	AT3G21770
BnaA07g32300D	Thioredoxin-like protein CDSP32, chloroplastic	1.26	0.001144	AT1G76080
Calcium ion				
BnaC04g34480D	Calcium-binding protein CML24	1.54	0.01832	AT5G37770
BnaC03g61330D	Calcium-transporting ATPase 2, plasma membrane-type	1.22	0.03131	AT4G37640
BnaC05g09500D	Probable calcium-binding protein CML13	0.81	0.01581	AT1G12310
BnaC04g34470D	Calmodulin-4	0.74	0.0003448	AT1G66410
BnaA03g39650D	Calcium sensing receptor, chloroplastic	0.63	0.02561	AT5G23060

In order of Mean Ratio (*inap*-vs-H3), and the DAPs were identified at level of *P*-value < 0.05.

Table S5. Primer sequences for the DAPs used in qRT-PCR assays and relative changes in expression.

DAPs ID	Description	Mean Ratio (iTRAQ)	Regulation (*inap* CMS vs H3)	Primer (5′–3′)
BnaA09g12770D	Elongation factor G, chloroplastic	1.53	Up	F-GAAGAGATTGGTGAGGAAAGGA<br>R-AGATAGTCAACCACAGCATCAA
BnaA07g08200D	Pollen-specific protein-like	0.48	Down	F-GTAACGTTGACTAAAGAAGCCG<br>R-CTAACGGCGTTTTAACCATGAA
BnaA03g58200D	Pollen-specific protein-like	0.37	Down	F-AAGAAGCTATGACCGACAAAGA<br>R-GTTAATGGTTCGAGTCTCGTTG
BnaC08g23600D	ATP synthase subunit d, mitochondrial	0.53	Down	F-GTTCAAAGCTTCAAGGACGATT<br>R-TTGTACATGTCAACAATGCCAG
BnaA10g24380D	D-lactate dehydrogenase [cytochrome], mitochondrial	0.65	Down	F-ACAACACGGACTATTCTTTCCT<br>R-CTCCATTAGGAAGAACCACCTT
BnaA05g29810D	Exopolygalacturonase clone GBGA483	0.63	Down	F-AACACCAACAACGATTACAGTG<br>R-GATTGCCATGCATATCGAAAGT
BnaA03g04340D	ATP synthase subunit O, mitochondrial	0.56	Down	F-GAGAGATTATGCAACAGCTTCG<br>R-CGAGTCTTGTCTCTCTAGGAAC
BnaC02g00700D	Probable pectinesterase/pectinesterase inhibitor 51	0.8	Down	F-CGGAGGAGTACATGAAACTGTA<br>R-ACTCTCCATAATACAGCGTCTG
BnaC01g39610D	Polygalacturonase	0.42	Down	F-GTTAAGAACTGTACCCTCGAGG<br>R-GGATTGGGTTGCTAACTTTGTT
BnaCnng35540D	Probable glucan endo-1, 3-beta-glucosidase A6	0.57	Down	F-GTTCTGTTCCTCTTCACTCTCA<br>R-CTACTTGCGAGTGATGACAATG
BnaA10g24240D	Peroxidase 53	0.61	Down	F-TCTTGATCTGAGCACACCTGAC<br>R-AGACTCTGGTTACTTGCGAAGG
BnaA03g39650D	Calcium sensing receptor, chloroplastic	0.63	Down	F-AATCAACAAAGGCTCCAACATC<br>R-AGAAGCTGCTGGTATAATCCTC
Actin				F-CTGTGCCAATCTACGAGGGT<br>R-CTTACAATTTCCCGCTCTGCT

Table S6. Comparison of gene and protein expression ratios between the transcriptomic (RNA-Seq) and proteomic (iTRAQ) analysis.

Gene/Protein ID	log_2(Fold Change) (0–5 DEGs)	log_2(Fold Change) (6–8 DEGs)	inap/H3@Mean Ratio (iTRAQ)	Annotation
BnaC08g42050D	-3.53		0.52	Thioredoxin domain-containing protein
BnaA09g18440D	-2.94		0.61	Acetyl-CoA acetyltransferase
BnaC07g25330D	-2.70		0.56	DUF1216 domain-containing protein
BnaA02g05310D	-2.66		0.69	AMPKBI domain-containing protein
BnaA06g31290D	-2.45		0.53	Uncharacterized protein
BnaC09g37560D	-2.39	-2.23	0.82	Phytocyanin domain-containing protein
BnaA10g04490D	-2.33		0.64	Uncharacterized protein
BnaA08g00900D	-2.18		0.72	Aldehyde dehydrogenase family 7 member A1
BnaC03g18820D	-2.15		0.46	Auxin-repressed 12.5 kDa protein
BnaC03g69760D	-2.14		0.75	Persulfide dioxygenase ETHE1 homolog, mitochondrial
BnaC03g40460D	2.06		2.09	Non uncharacterized protein specific lipid
BnaC03g36340D	2.12		1.56	Class-III pyridoxal-phosphate-dependent aminotransferase
BnaC01g30760D	2.23		2.01	Uncharacterized protein
BnaC06g34250D	2.24		2.48	Protease inhibitor I3 family
BnaC02g00130D	2.32		0.67	Protein aspartic protease in guard cell 2
BnaAnng00260D	2.33		1.48	Aspartic proteinase PCS1
BnaC04g39350D	2.38		1.29	Histone deacetylase HDT4
BnaC01g31810D	2.48	2.04	1.75	Peroxidase 30
BnaC03g06120D	2.55	2.01	1.5	Chalcone synthase 3
BnaA02g05650D	2.95		1.73	Uncharacterized protein
BnaC06g12160D	3.79		1.37	Diacylglycerol O-acyltransferase WSD1
BnaA04g22080D	3.87	2.67	1.62	Non-specific lipid-transfer protein A
BnaCnng01440D	3.94	4.26	1.64	Pept_C1 domain-containing protein
BnaA02g12440D	4.21	4.65	1.4	Glycosyl hydrolase 17 family
BnaC06g10100D	4.22		1.45	WD40 repeat
BnaA10g22800D	4.35		1.25	Flavonol synthase/flavanone 3-hydroxylase
BnaC06g37960D	4.94	2.45	1.43	Probable acyl Uncharacterized protein activating enzyme 4
BnaA06g35420D		-2.29	0.32	14.7 kDa heat shock protein
BnaA09g53230D		2.39	1.35	ADP-ribosylation factor GTPase-activating protein AGD2
BnaA01g08120D		2.41	1.62	GDSL esterase/lipase
BnaA05g25900D		-3.45	0.53	L-ascorbate oxidase homolog
BnaA02g16610D		2.77	0.83	Uridine-cytidine kinase C
